# Proposal of ‘reactive‐lymphocyte/histiocyte rich large B‐cell lymphoma’ as an alternate term for ‘nodular lymphocyte predominant Hodgkin lymphoma’ that would also address its overlap with T‐cell/histiocyte rich large B‐cell lymphoma

**DOI:** 10.1002/jha2.560

**Published:** 2022-08-31

**Authors:** Kikkeri N. Naresh

**Affiliations:** ^1^ Section of Pathology, Clinical Research Division Fred Hutchinson Cancer Research Center Seattle Washington USA; ^2^ Department of Laboratory Medicine and Pathology University of Washington Seattle Washington USA

1

In the WHO classification, Hodgkin lymphoma is broadly classified as nodular lymphocyte predominant Hodgkin lymphoma (NLPHL) and classic Hodgkin lymphoma (CHL) [1]. While this approach has served us well for many years, there is renewed thinking as to whether ‘NLPHL’ should be excluded from Hodgkin lymphoma. The reasoning comes from – (1) the overlap and biological relatedness to T‐cell/histiocyte rich large B‐cell lymphoma (THRLBCL); and (2) the distinctiveness of the neoplastic cells of NLPHL from CHL [2]. If these reasons are valid, what would be the appropriate term that would address the issues and capture the characteristic features?

NLPHL shares two features with CHL – (1) neoplastic cells represent a small minority of all cells, with the rest being benign/reactive cells; (2) the neoplastic cells are large with lobulated or multilobated nuclei. However, there are important differences. The B‐cell program is fully maintained in NLPHL, whereas the B‐cell program is heavily suppressed in CHL. Furthermore, relapses of CHL continue to have features of CHL, while relapses of NLPHL can take features of THRLBCL or rarely of diffuse large B‐cell lymphoma (DLBCL). In large studies, patients with THRLBCL have been shown to have an 18.2‐fold higher association with prior NLPHL as compared to those with DLBCL supporting a strong link between NLPHL and THRLBCL. Furthermore, outcomes of patients with THRLBCL are significantly better than DLBCL [3].

Our current understanding of the immunohistological features of NLPHL is that the neoplastic cells are of germinal center‐derived B‐cell origin with a well‐maintained B‐cell program. The lesion is composed of scattered large neoplastic B‐cells with multilobated nuclei amidst abundant reactive small lymphocytes with or without histiocytes, and the lesion has a nodular pattern at least in parts. NLPHL can present with six growth patterns (Fan patterns, A–F) [4]. Typical NLPHL has a prominent nodular pattern and a predominant background of reactive small B‐cells (patterns A and B; 60%–75% of cases) [4, 5]. Rest has either a predominantly diffuse pattern and/or marked depletion of small reactive B‐cells along with predominance of small reactive T‐cells (variant patterns C–F). Patients with variant patterns particularly those with depletion of small reactive B‐cells and predominance of small reactive T‐cells have a higher frequency of advanced stage disease and higher relapse rates [6]. The distinction of T‐cell rich variant patterns, particularly pattern ‘E’ from THRLBCL depends on the recognition of at least one unequivocal NLPHL nodule, which in turn depends on the type/size of the biopsy. Hence, in limited biopsies, NLPHL cases with reactive T‐cell rich variant patterns can be difficult to distinguish from THRLBCL. It should also be noted that more than one pattern of NLPHL can co‐exist in a single case; for example, in the original study of Fan et al., pattern E was a minor component in 19 of 35 cases with pattern E [4]. Furthermore, more than one‐half of patients of NLPHL can switch to a variant histology at relapse [7]. NLPHL has some morphological overlap with lymphocyte rich CHL (LRCHL); however, the neoplastic cells of NLPHL differ from those in LRCHL by complete preservation of the B‐cell program [8].

THRLBCL is as an aggressive B‐cell lymphoma with <10% large neoplastic B‐cells, that are scattered in a diffuse background rich in T‐cells and histiocytes, and almost no small B‐cells. It is acknowledged that THRLBCL has clinical, immunophenotypic, transcriptomic, and genetic overlap with NLPHL [9, 10]. Patients with THRLBCL often present in stages III or IV.

Two issues need to be addressed – (1) if NLPHL is not the appropriate term, what should this be replaced with? (2) what would be a pragmatic approach to address the overlap of NLPHL with THRLBCL? A recent publication has suggested the term ‘nodular lymphocyte predominant B‐cell lymphoma (NLPBL)’ to replace NLPHL [11]. The term NLPBL implies that the lymphoma is – (1) B‐cell in origin, (2) nodular in pattern and (3) has predominance of lymphocytes. The term fails to capture the larger size and the unusual cytological characteristics of the neoplastic B‐cells, the reactive nature of the lymphocytes, and the lack of an obvious nodular pattern in some cases. Furthermore, there are many other B‐cell lymphomas that have a nodular pattern and show predominance of lymphocytes. Although the 5th edition of the WHO classification preserves NLPHL in its current form, the term NLPBL has been cited as an acceptable alternate term [1, 12]. It would be appropriate to propose and explore alternate concepts and strategies and discuss these in open forums ahead of the next (6th) edition of the WHO classification.

Ideally, the replacement term for NLPHL should document the large B‐cell nature of the neoplastic cells, and the abundance and the nature of the reactive cells. Additionally, it should accommodate the immunohistological heterogeneity and reconcile the overlap with THRLBCL. To address this, I propose the term ‘reactive‐lymphocyte/histiocyte rich large BCL (RLHRLBL)’ with three grades – grade 1 (prominent reactive small B‐cells with a nodular pattern) and grades 2 and 3 (lack of a nodular pattern and/or depletion of reactive small B‐cells). Grade 1 would incorporate the current NLPHL with Fan patterns A and B, while grade 2 will incorporate NLPHL with Fan patterns C–F, and grade 3 would represent THRLBCL. This would address both the immunohistological heterogeneity of NLPHL and the overlap with THRLBCL and present the disease process as a continuum. In RLHRLBCL, small reactive lymphoid cells can be either B‐cells or T‐cells. With increasing disease aggressiveness (associated with proportionally unfavorable outcomes), the proportion of reactive B‐cells and follicular dendritic cells (nodular pattern) decrease in the milieu, and the proportion of reactive T‐cells increase. Under such a scheme, RLHRLBL grade 1 can progress or transform to RLHRLBL grade 2 or grade 3, or to DLBCL (Figure 1).

**FIGURE 1:**
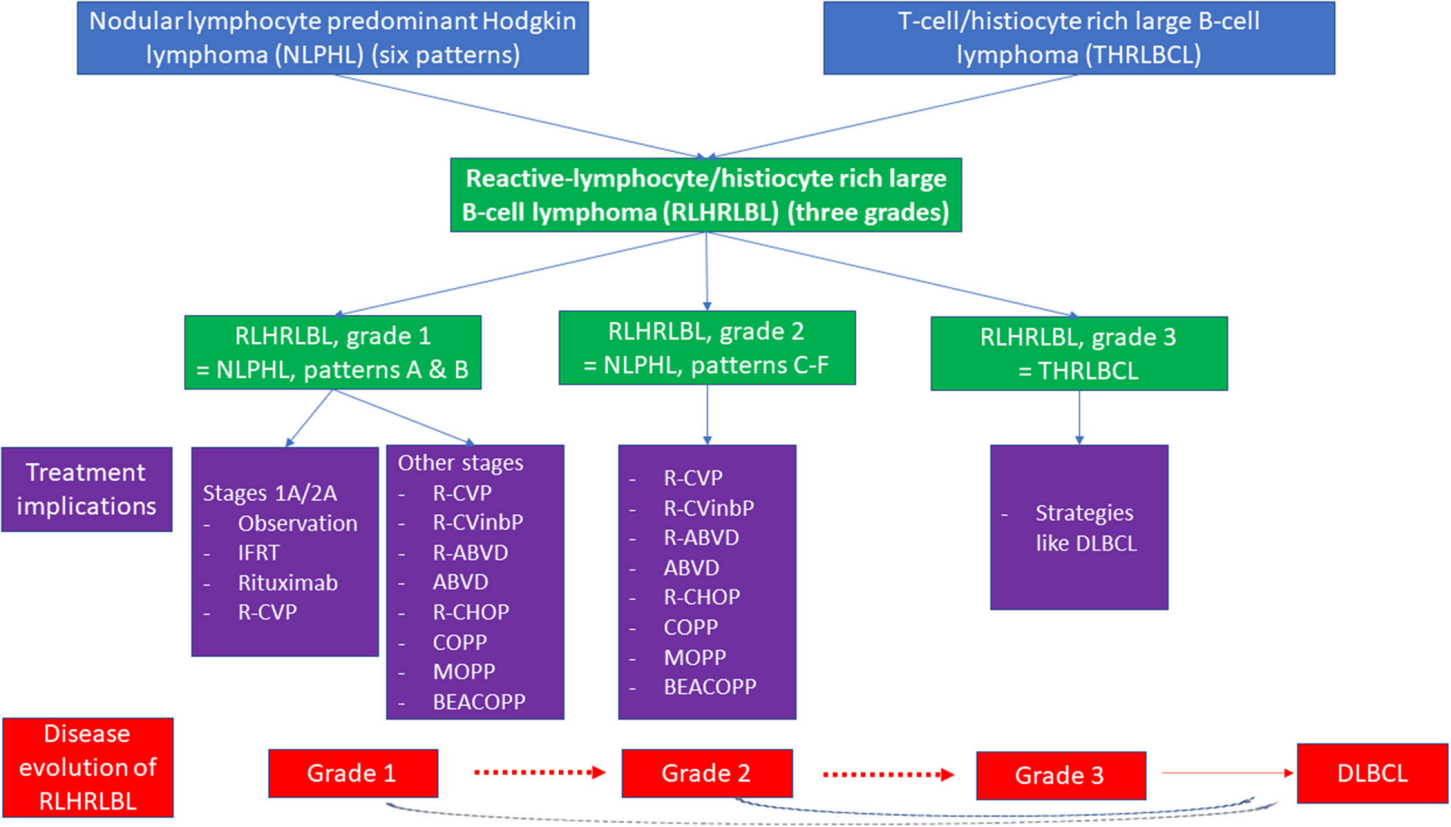
Proposal of a new lymphoma term/entity ‘reactive‐lymphocyte/histiocyte rich large B‐cell lymphoma (RLHRLBL)’: The new term ‘Reactive‐lymphocyte/histiocyte rich large B‐cell lymphoma (RLHRLBL)’ is proposed to amalgamate the terms/entities nodular lymphocyte predominant Hodgkin lymphoma (NLPHL) and T‐cell/histiocyte rich large B‐cell lymphoma (THRLBCL). RLHRLBL will have three grades: grade 1 replacing NLPHL of patterns A and B; grade 2 replacing NLPHL of patterns C–F; and grade 3 replacing THRLBCL. Treatment implications of such a system are shown. The disease continuum (grade 1 to 3) also captures disease evolution in a minority of patients with a small minority of patients evolving to DLBCL.

Such an approach may help stratify patients for treatment: (1) low‐stage disease (I and II) without any B symptoms, and grade 1 histology ‐ observation/involved‐field‐radiotherapy/Rituximab/R‐CVP; (2) high‐stage disease, B‐symptoms or grade 2 histology ‐ R‐CVP/R‐CVinbP/R‐ABVD/ABVD/R‐CHOP/COPP/MOPP/BEACOPP; and (3) grade 3 histology ‐ lines similar to DLBCL [13, 14].

## AUTHOR CONTRIBUTION

KNN conceptualized and wrote the manuscript.

## CONFLICT OF INTEREST

The author declares that there is no conflict of interest that could be perceived as prejudicing the impartiality of the research reported.

## FUNDING INFORMATION

The author received no specific funding for this work.

## Data Availability

Data sharing not applicable to this article as no datasets were generated or analysed during the current study.
